# The evolution of the initial manifestations and renal involvement of chinese patients with classical and late-onset Fabry disease at different sexes and ages

**DOI:** 10.1186/s12882-023-03138-w

**Published:** 2023-04-05

**Authors:** Wenkai Guo, Yuansheng Xie, Pengcheng Ji, Shuang Li, Guangyan Cai, Xiangmei Chen

**Affiliations:** 1 School of Medicine, Nankai University, Tianjin, China; 2 Department of Nephrology, First Medical Center of Chinese PLA General Hospital, Chinese PLA Institute of Nephrology, State Key Laboratory of Kidney Diseases, National Clinical Research Center for Kidney Diseases, Beijing, China

**Keywords:** Fabry disease, Initial manifestation, Renal involvement, Classical phenotype, Evolution

## Abstract

**Background:**

Fabry disease is a rare hereditary disease involving multiple organs, and there are few reports on how the initial manifestations and renal involvement of these patients with classical and late-onset phenotype evolve with sexes and ages. To improve clinicians’ understanding of Fabry disease and avoid misdiagnoses by discussing the initial manifestations, first medical specialties visited and renal involvement development in patients.

**Methods:**

This study collected relevant data from 311 Chinese Fabry disease patients (200 males, 111 females) and descriptive statistical analysis was used to analyze the evolution of the initial manifestations and renal involvement of patients with classical and late-onset phenotype at different sexes and ages.

**Results:**

Regarding the age at manifestation onset, age at the first medical specialty visited and age at the diagnosis of Fabry disease, males were earlier than females, and males with classical phenotype were earlier than males with late-onset and females with classical phenotype. In both male and female patients, the initial manifestations of classical patients were mainly acroparesthesia, and the first medical specialty visited were mainly pediatrics and neurology. The initial manifestations of late-onset patients were mainly renal and cardiovascular involvement, and the first medical specialty visited were mainly nephrology and cardiology. In classical patients, both male and female, the initial manifestations of the preschool and the juvenile groups were mainly acroparesthesia, and the frequency of renal and cardiovascular involvement in the young group was higher than that in the preschool and juvenile groups. There was no obvious renal involvement in the preschool group, renal involvement was most common in the young group and the middle-aged and elderly group. Proteinuria can appear in classical male patients as early as approximately 20 years, and renal insufficiency can occur at approximately 25 years. With age, over 50% of classical male patients can develop varying degrees of proteinuria at the age of 25 and renal insufficiency at the age of 40. 15.94% of the patients progressed to dialysis or kidney transplantation, mainly classical males.

**Conclusions:**

The initial manifestation of Fabry disease is affected by sex, age and classical/late-onset phenotype. The initial manifestations were mainly acroparesthesia and the frequency and degree of renal involvement increased gradually with aging in classical male patients.

**Supplementary Information:**

The online version contains supplementary material available at 10.1186/s12882-023-03138-w.

## Background

Fabry disease is a rare X-linked hereditary lysosomal storage disorder [1, 2] caused by mutations in the *GLA* gene located on Xq22.1 encoding the lysosomal enzyme alpha-galactosidase A (α-Gal A). Mutations in the *GLA* gene lead to the absence or deficiency of α-Gal A and the consequent accumulation of glycosphingolipids, such as globotriaosylceramide (Gb3) and its deacylated derivative globotriaosylsphingosine (lyso-Gb3), within tissues. This accumulation of glycosphingolipids results in cell dysfunction, which progressively affects multiple organ systems [3, 4], thereby leading to a series of clinical manifestations [5–7], such as acroparesthesia, angiokeratomas, sweating abnormalities, gastrointestinal manifestations, and eye and ear lesions. As the disease progresses, multiorgan damage is gradually aggravated, which can eventually lead to life-threatening kidney, heart and cerebrovascular system complications and even premature death [8–10]. Meanwhile, Fabry disease can be divided into classical and late-onset phenotype according to characteristic symptoms and enzyme activity. Patients may present a series of clinical manifestations, ranging from severe classical phenotype in males to asymptomatic in some females, as well as various clinical manifestations of multi-organ involvement in between of them [11].

Due to the heterogeneity, diversity and complexity of the clinical manifestations of Fabry disease, as well as the imperfect disease screening system, patients in the early stage of the disease are easily misdiagnosed and even mistreated. For most patients, there is generally a long delay from the initial manifestations to the final diagnosis, and even adult patients may be misdiagnosed when complications such as those in the heart, brain and kidneys occur. Because of the misdiagnosis of Fabry disease as other diseases, some patients also receive unnecessary and inappropriate treatment, such as hormone and immunosuppressive therapy and even surgical treatment. Therefore, it is necessary to improve the current understanding of Fabry disease, to understand how the initial manifestations evolve in patients with sexes and ages and to understand which medical specialties patients visit. When patients begin to show clinical manifestations and signs of Fabry disease, it is of great importance to make an accurate diagnosis and to provide specific personalized treatment, especially enzyme replacement therapy (ERT) and chaperone therapy [12, 13], which has made Fabry disease one of the few rare genetic diseases that can be effectively treated at present.

Among the multiple organs involved in Fabry disease, progressive impairment of the kidneys is a pervasive clinical manifestation and is a common cause of premature death in patients. The accumulation of Gb-3 in glomeruli can occur in patients during the fetal period. Pathological changes in the kidneys can also be observed in renal biopsy samples from some children or adolescent patients who have not yet developed renal manifestations (such as proteinuria and decreased glomerular filtration rate) [14, 15]. Almost all classical male hemizygous and some female heterozygous patients will have varying degrees of renal involvement, and with age, renal function will gradually deteriorate and eventually progress to end-stage renal disease (ESRD), with renal replacement therapy being required in severe cases [14]. However, there are currently few studies on the occurrence and development of renal involvement in Fabry disease. Therefore, understanding how renal involvement evolves in Fabry disease can help better identify the disease earlier.

Previous clinical studies of Fabry disease were mostly performed in Europe and the United States, while studies on large samples from Asian populations, especially the Chinese population, are relatively rare, and most reported cases in this population are sporadic clinical cases. Due to the large total population of China, rare diseases are not relatively uncommon in China. As a rare disease with a relatively high prevalence, Fabry disease has been listed as one of the 121 rare diseases identified in China in 2018. This study is currently the largest clinical study of Fabry disease in China, and its aim is to improve the understanding and diagnosis of Fabry disease, shorten the time to diagnosis, and reduce the rates of misdiagnosis by exploring how the initial manifestations of patients evolve with age and the visited medical specialties and renal involvement of 311 patients with classical and late-onset Fabry disease at different sexes and ages.

## Methods

### Study population

In this study, the accumulation of medical records (311 patients diagnosed with Fabry disease) was from June 2012 to May 2022. A questionnaire survey was conducted on patients in April 2022, and the statistical analysis was conducted based on the data of the questionnaire survey. The inclusion criteria for these patients refer to the consensus recommendation of treatment experts for the diagnosis of Fabry disease [4, 16] based on the presence of typical clinical manifestations, a family history, laboratory examination results (*GLA* gene analysis, α-Gal A activity and the concentration of the enzyme substrate Lyso-Gb3), and pathological changes (in kidney, heart muscle, nerve, or skin biopsy samples) for comprehensive judgment. This clinical study was reviewed and approved by the Medical Ethics Committee of the Chinese PLA General Hospital (approval number: 2012-001), and all procedures of this clinical study were fully in accordance with the principles of the Declaration of Helsinki. All participants provided written informed consent. For minors, parents/legal guardians were informed, and their parents/legal guardians signed the informed consent instead.

### Clinical data collection

In this clinical study, outpatients, previously hospitalized patients and Fabry patients enrolled in National Rare Disease Registry System of China were selected as research objects, and the clinical data of patients were further obtained through questionnaire survey. These data included general information (sex, age, family history and so on), clinical data (initial manifestations, age at the onset of clinical manifestations, age at first visit, age at diagnosis, first medical specialty visited, final diagnosis medical specialty, season of onset, and clinical manifestations of various involved systems, including the urinary system, cardiovascular system, neuropsychiatric system, digestive system, eyes, ears and so on) and laboratory results (α-galactosidase A activity was detected by the dried blood spot or fluorescent substrate methods, and *GLA* gene analysis was performed by next-generation sequencing).

### Clinical grouping and definition of clinical indicators

Patients were divided into male and female groups according to sex and into a preschool group (< 7 years), juvenile group (≥ 7 years and ≤ 18 years), young group (> 18 years and ≤ 45 years) and middle-aged and elderly group (> 45 years) according to age. Patients were classified as classical or nonclassical (late-onset) Fabry disease on the basis of their *GLA* gene mutation, enzyme activity (men only), and the presence or absence of characteristic organ involvement manifestations.

The criteria for the judgment of classical and late-onset Fabry disease [11]: males with Fabry disease were considered to have a classical phenotype when they met all of the following criteria:1. a *GLA* mutation, 2. α-Gal A activity ≤ 5% of the mean value of the normal reference range, and 3. the presence of one or more of the characteristic manifestations of Fabry disease (neuropathic pain of Fabry, angiokeratoma, and/or corneal vortex-like opacity). Males with Fabry disease who did not meet these criteria were categorized as late-onset Fabry disease. Female patients with Fabry disease were considered to be classical when they had *GLA* mutations and one or more characteristic manifestations of Fabry disease, while female patients without characteristic manifestations of Fabry disease were classified as late-onset.

Indicator criteria: 1. Renal involvement includes proteinuria, renal insufficiency and ESRD. Proteinuria: quantitatively, urine protein > 150 mg/24 h or qualitatively, urine positive for protein; Renal insufficiency: serum creatinine exceeding the upper limit of normal value of the local hospital; ESRD: chronic kidney disease (CKD) stage 5, glomerular filtration rate < 15 mL/min/1.73 m^2^ or dialysis or kidney transplantation [17]. 2. Cardiovascular involvement: the presence of any of the following manifestations can be considered cardiovascular involvement: left ventricular hypertrophy, arrhythmia, classical Fabry disease ECG findings.

### Statistical analysis

Data analysis was performed using the statistical analysis software SPSS 26.0, and graphing was performed using GraphPad Prism 9.3.0. Data with a normal distribution are expressed as the mean ± standard deviation (SD) and were compared by t-tests. Measurement data that did not conform to a normal distribution are expressed as the median (interquartile range) [M (P25, P75)]. Differences were compared using the rank sum test and Kruskal-Wallis test with K independent samples. Categorical variables are expressed as rates (%), and the difference between two groups was compared using the chi-square test. Differences between multiple groups were compared using the chi-square test or Fisher’s exact probability test. Multiple comparisons were made using the Bonferroni method. *P* < 0.05 was considered statistically significant.

## Results

### Analysis of geographical distribution, onset season, gene test, enzyme activity test and histopathologic biopsy of patients with Fabry disease

In this study, the geographical distribution of 311 patients with Fabry disease was analyzed, and the results showed that a total of 309 patients provided geographical distribution data. Except for Hong Kong, Macao, Taiwan and Ningxia, patients with Fabry disease have spread across 30 provinces and autonomous regions in China. Fabry disease patients were mainly concentrated in East China (101 cases, 32.69%), Central China (67 cases, 21.68%) and North China (61 cases, 19.74%). Subdivided into provinces and cities, the top five provinces in terms of the proportion of Fabry disease patients are Henan (34 cases), Jiangxi (29 cases), Shanxi (27 cases), Hebei (25 cases) and Hunan (24 cases), as shown in Figure S1 in supplementary material.

In terms of the onset season, summer had the largest proportion of patients with onset up to 58.70%, followed by winter (16.67%), spring (12.32%) and autumn (12.32%), with no significant difference between sexes in the onset season.

Of the 311 patients with Fabry disease included in this study, 163 underwent *GLA* gene testing, the results of *GLA* gene mutation analysis showed that missense mutation was the most common mutation type (61.96%), followed by nonsense mutation (16.56%), frameshift mutation (13.50%), splice site mutation (4.91%) and large fragment deletion (3.07%).

170 underwent α-Gal A activity testing, which show that the activity of α-Gal A in classical patients was significantly lower than that in patients with late-onset phenotype [0.29 (0.20, 0.36) vs. 2.90 (0.89, 4.66) µmol/L/h (dried blood spot). 0.52 (0.30, 11.54) vs. 4.45 (0.85,24.33) nmol/g/min (fluorescent substrate method)], and the activity of α-Gal A in males was significantly lower than that in females [0.29 (0.22, 0.35) vs. 3.57 (1.53, 5.15) µmol/L/h (dried blood spot), 0.40 (0.20, 0.70) vs. 25.35 (16.05, 35.78) nmol/g/min (fluorescent substrate method)].

81 underwent histopathological biopsies (kidney, heart, skin, or nerve tissue biopsies), of which 75 patients received renal biopsy. A total of 96.24% of the patients had a family history of Fabry disease. Among the family members, 50.51% had end-stage renal disease, 55.56% had heart disease, and 32.32% had stroke.

### Comparison of the age at onset, first visit and final diagnosis, the initial manifestations, first medical specialty visited and final diagnosis medical specialty of patients with classical and late-onset Fabry disease at different sexes

A total of 311 patients with Fabry disease were included, including 200 males (64.31%) and 111 females (35.69%). According to the clinical phenotypes, they were divided into classical (237 cases, 76.21%) and late-onset phenotype (74 cases, 23.79%).

Regarding the age at manifestation onset, age at the first medical specialty visited and age at the diagnosis of Fabry disease, males were earlier than females, and males with classical phenotype were earlier than males with late-onset and females with classical phenotype. There was no significant difference between late-onset males and females. The average interval from the initial onset of clinical manifestations to the final diagnosis were 14.48 ± 11.85 years.

Among the initial manifestations of Fabry disease in males and females, the top five were acroparesthesia (limb pain), hypohidrosis/anhidrosis, renal involvement, cardiovascular involvement and angiokeratomas. The incidence rate of acroparesthesia as the initial manifestation in males was significantly higher than that in females, and the frequency of initial manifestation of cardiovascular involvement in females was significantly higher than that in males. In both male and female patients, the initial manifestations of classical patients were mainly acroparesthesia, and the initial manifestations of late-onset patients were mainly renal and cardiovascular involvement.

In both male and female patients, the first medical specialty visited of classical patients were mainly pediatrics and neurology and the first medical specialty visited of late-onset patients were mainly nephrology and cardiology. The top five definite diagnosis medical specialties were the nephrology, neurology, pediatrics, genetics and cardiology (see Table 1).

**Table 1:** Comparison of the age at onset, first visit and final diagnosis, the initial manifestations, first visit and final diagnosis medical specialty of patients with classical and late-onset Fabry disease at different sexes

	All patients (N = 311)	Males (n = 200)	Classical males (172)	Late-onset males (28)	Females (n = 111)	Classical females (65)	Late-onset females (46)
**Age at onset (years)**	14.69 ± 11.96	12.69 ± 9.10	11.32 ± 7.72	27.67 ± 9.77^**a**^	18.99 ± 15.72 ^**b**^	17.53 ± 15.64	22.95 ± 15.61
**Age at first visit (years)**	19.63 ± 12.80	17.55 ± 10.60	16.10 ± 9.75	30.93 ± 8.80 ^**a**^	24.98 ± 16.11 ^**b**^	23.17 ± 16.15	32.25 ± 14.36
**Age at final diagnosis (years)**	29.55 ± 12.32	27.46 ± 9.77	26.90 ± 9.63	33.94 ± 8.31^**a**^	34.71 ± 16.03 ^**b**^	34.58 ± 16.53	35.21 ± 14.42
**Initial manifestations [n/N (%)]**							
Acroparesthesia (limb pain)	186 /296 (62.84)	138/196 (70.41)	138/171 (80.71)	0/25 ^**a**^	48 /100 (48.00) ^**b**^	47/64 (73.44)	1/36 (2.78) ^**c**^
Anhidrosis or hypohidrosis	47 /296 (15.88)	32 /196 (16.33)	29/171 (17.00)	3/25 (12.00)	15 /100 (15.00)	11/64 (17.19)	4/36 (11.11)
Renal involvement	46 /296 (15.54)	33 /196 (16.84)	15/171 (8.77)	18/25 (72.00) ^**a**^	13 /100 (13.00)	2/64 (3.13)	11/36 (30.56) ^**c**^
Cardiovascular involvement	18 /296 (6.08)	13 /196 (6.63)	13/171 (7.60)	0/25	5 /100 (5.00)	5/64 (7.81)	0/36
Angiokeratomas	19 /296 (6.42)	6 /196 (3.06)	3/171 (1.75)	3/25 (12.00) ^**a**^	13 /100 (13.00) ^**b**^	3/64 (4.69)	10/36 (27.78) ^**c**^
Neuropsychiatric involvement	8 /296 (2.70)	1 /196 (0.51)	1/171 (0.58)	0/25	7 /100 (7.00) ^**b**^	1/64 (1.56)	6/36 (16.67) ^**c**^
Digestive system involvement	6 /296 (2.03)	1 /196 (0.51)	0/171	1/25 (4.00)	5 /100 (5.00) ^**b**^	4/64 (6.25)	1/36 (2.78)
Others manifestations	3 /296 (1.01)	1/196 (0.51)	0/171	1/25 (4.00)	2 /100 (2.00)	2/64 (3.13)	0/36
**The first medical specialty visited [n/N (%)]**							
Pediatric	66 /249 (26.51)	51 /172 (29.65)	51/152 (33.55)	0 /20 ^**a**^	15 /77 (19.48)	13/56 (23.21)	2/21 (9.52)
Neurology	49 /249 (19.68)	34 /172 (19.77)	34/152 (22.37)	0/20 ^**a**^	15 /77 (19.48)	14/56 (25.00)	1/21 (4.76) ^**c**^
Nephrology	46 /249 (18.47)	35 /172 (20.35)	18/152 (11.84)	17/20 (85.00) ^**a**^	11 /77 (14.29)	4/56 (7.14)	7/21 (33.33) ^**c**^
Rheumatology and Immunology	23 /249 (9.24)	16 /172 (9.30)	16/152 (10.53)	0 /20	7 /77 (9.09)	7/56 (12.50)	0/21
Cardiovascular	15 /249 (6.02)	4 /172 (2.33)	3/152 (1.97)	1/20 (5.00)	11 /77 (14.29) ^**b**^	3/56 (5.36)	8/21 (38.10) ^**c**^
Dermatology	13/ 249 (5.22)	7 /172 (4.07)	7/152 (4.61)	0/20	6 /77 (7.79)	4/56 (7.14)	2/21 (9.52)
Pain management clinic	9 /249 (3.61)	7 /172 (4.07)	7/152 (4.61)	0/20	2 /77 (2.60)	2/56 (3.57)	0/21
Orthopaedics	5 /249 (2.01)	5 /172 (2.91)	5/152 (3.29)	0/20	0/77	0/56	0/21
Gastroenterology	4 /249 (1.61)	2 /172 (1.16)	1/152 (0.66)	1/20 (5.00)	2 /77 (2.60)	2/56 (3.57)	0/21
Other medical specialties	19 /249 (7.63)	11 /172 (6.40)	11/152 (7.24)	0/20	8 /77 (10.39)	7/56 (12.50)	1/21 (4.76)
**The final diagnosis medical specialty [n/N(%)]**							
Nephrology	121 /217 (55.76)	93 /154 (60.39)	79/139 (56.83)	14/15 (93.33) ^**a**^	28 /63(44.44)	18/50 (36.00)	10/13 (76.92) ^**c**^
Neurology	42 /217 (19.35)	28 /154 (18.18)	28/139 (20.14)	0/15	14 /63(22.22)	14/50 (28.00)	0/13 ^**c**^
Pediatric	20 /217 (9.22)	13 /154 (8.44)	13/139 (9.35)	0/15	7 /63(11.11)	7/50 (14.00)	0/13
Medical Genetics	13 /217 (5.99)	6 /154 (3. 90)	6/139 (4.32)	0/15	7 /63(11.11) ^**b**^	6/50 (12.00)	1/13 (7.69)
Cardiology	11 /217 (5.07)	8 /154 (5.19)	7/139 (5.04)	1/15 (6.67)	3 /63(4.76)	1/50 (2.00)	2/13 (15.38) ^**c**^
Rheumatology and Immunology	4 /217 (1.84)	1 /154 (0.65)	1/139 (0.72)	0/15	3 /63(4.76) ^**b**^	3/50 (6.00)	0/13
Dermatology	1 /217 (0.46)	1 /154 (0.65)	1/139 (0.72)	0/15	0/63	0/50	0/13
Other medical specialties	5 /217 (2.30)	4 /154 (2.60)	4/139 (2.88)	0/15	1 /63 (1.59)	0/50	1/13 (7.69) ^**c**^

Note: ^**a**^ late-onset male patients compared with classical patients, the difference was statistically significant (*P* < 0.05); ^**b**^ Female patients compared with male patients, the difference was statistically significant (*P* < 0.05); ^**c**^ late-onset female patients compared with classical patients, the difference was statistically significant (*P* < 0.05)

### Comparison of the initial manifestations of patients with classical and late-onset Fabry disease at different sexes and ages

A total of 254 patients with Fabry disease who had complete information on initial manifestations were divided into male (176 patients) and female (78 patients) groups according to sex, divided into classical (219 patients) and late-onset phenotype (35 patients) according to their clinical phenotypes and were further divided into four groups according to the age at initial onset: preschool group (≤ 7 years), juvenile group (> 7 years and ≤ 18 years), young group (> 18 years and < 45 years) and middle-aged and elderly group (≥ 45 years).

In classical patients, both male and female, the initial manifestations of the preschool and juvenile groups were mainly acroparesthesia, and the frequency of renal and cardiovascular involvement in the young group was higher than that in the preschool and juvenile groups, see Table 2. Renal involvement was predominant in young male patients with late-onset. Due to the small sample size of patients with late-onset, especially after being dispersed to different sexes and age groups, the sample size of each group was even smaller, and there was no significant statistical difference in results, as shown in Table S1 in supplementary material.

**Table 2:** Comparison of the initial manifestations of classical patients with Fabry disease at different sexes and ages [N = 219, n/N (%)]

	Males(n = 161)				Females(n = 58)			
	Preschool group (n = 43)	Juvenile group (n = 100)	Young group (n = 18)	Middle-aged / elderly group (n = 0)	Preschool group (n = 8)	Juvenile group (n = 32)	Young group (n = 14)	Middle-aged / elderly group (n = 4)
**Acroparesthesia (limb pain)**	36/43 (83.72)	92/100 (92.00)	3/18 (16.67) ^**a, b**^	/	5/8 (62.50)	29/32 (90.63)	7/14 (50.00) ^**b**^	1/4(25.00) ^**b**^
**Anhidrosis/ hypohidrosis**	12/43 (27.91)	13/100 (13.00)	0/18 ^**a**^	/	2/8 (25.00)	2/32 (6.25)	3/14 (21.43) ^**b**^	0/4
**Renal invo**	1/43 (2.33)	3/100 (3.00)	12/18 (66.67) ^**a, b**^	/	0/8	0/132	1/14 (7.14)	0/4
**Cardiovascular invo**	0/43	0/100	4/18 (22.22) ^**a, b**^	/	0/8	0/32	2/14 (14.29) ^**b**^	1/4(25.00) ^**b**^
**Angiokeratomas**	2/43 (4.65)	6/100 (6.00)	1/18 (5.56)	/	0/8	1/32 (6.45)	3/14 (21.43) ^**b**^	0/4
**Neuropsychiatric invo**	0/43	1/100 (1.00)	0/18	/	0/8	0/32	0/14	1/4(25.00) ^**b**^
**Digestive system invo**	0/43	0/100	0/18	/	1/8 (12.50)	3/32 (9.38)	0/14	0/4
**Others manifestations**	0/43	0/100	0/18	/	0/8	1/32 (6.45)	0/14	1/4(25.00)

Abbreviations: invo, involvement; Note: ^**a**^ Compared with the preschool group, there is a statistical difference (*P* < 0.05); ^**b**^ Compared with the juvenile group, there is a statistical difference (*P* < 0.05)

### Comparison of renal involvement in patients with classical and late-onset Fabry disease at different sexes and ages

The common manifestations of renal involvement in both males and females were proteinuria and renal insufficient and ESRD. A total of 232 patients with Fabry disease had documented " renal involvement " in this study. The urinary data of Fabry patients were statistically analyzed. The patients were divided into classical (205 patients) and late-onset phenotype (27 patients), male (165 cases) and female (67 cases) groups and further divided into preschool, juvenile, young, middle-aged and elderly groups.

**Fig. 1:**
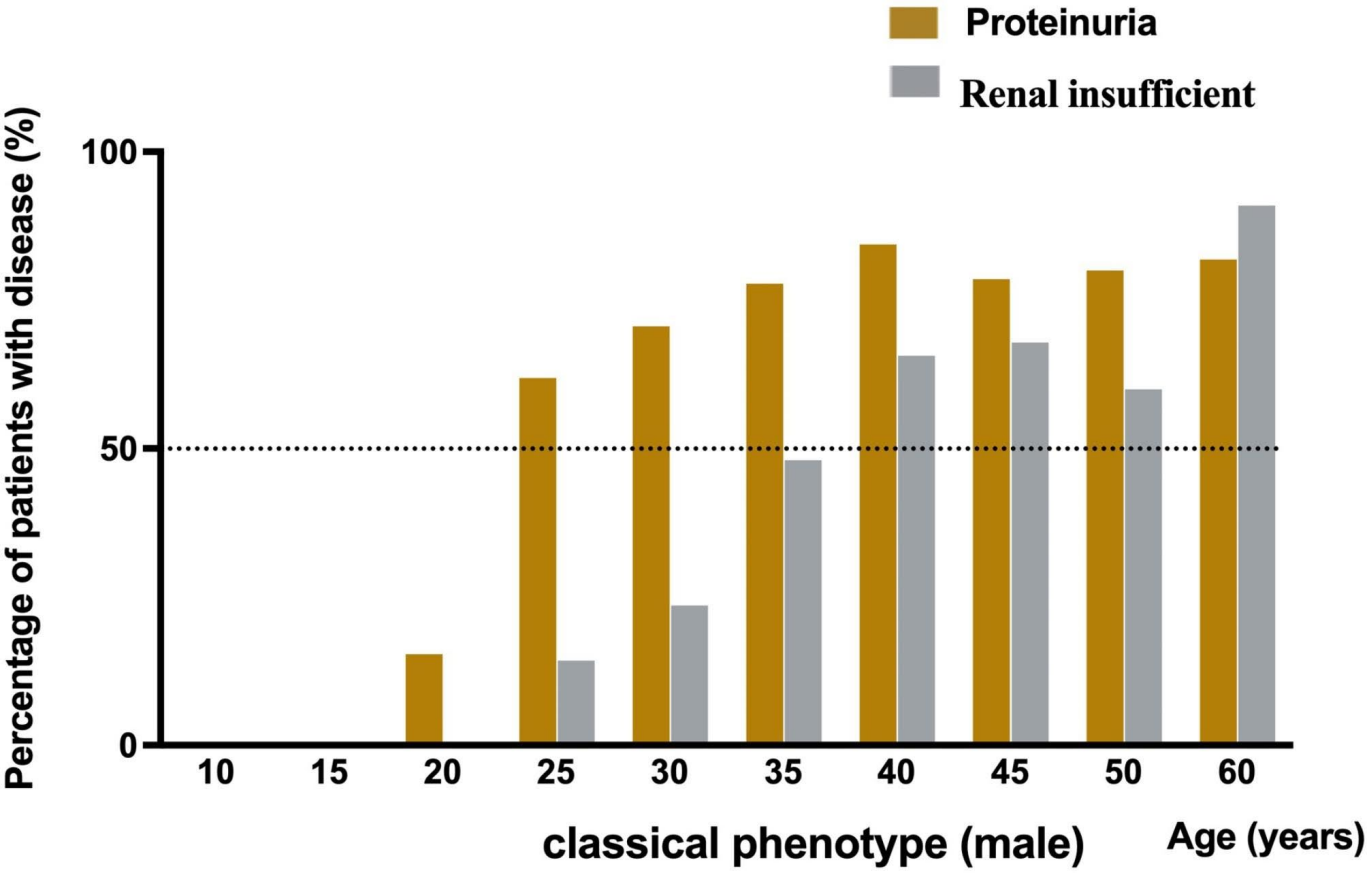
**Evolution of renal involvement in classical male Fabry disease patients with aging**. More than 50% of males with classical Fabry disease develop proteinuria of varying degrees at age 25 and renal insufficiency at age 40

In classical patients, both male and female, there was no obvious renal involvement in the preschool group, renal involvement was most common in the young group and middle-aged/ elderly group, see Table 3. The results of this analysis indicated that proteinuria can appear in classical male patients as early as approximately 20 years, and renal insufficiency can occur at approximately 25 years. With age, more than 50% of classical male patients can develop varying degrees of proteinuria at the age of 25 and renal insufficiency at the age of 40, as shown in Fig. 1. The evolution of renal involvement with age was not obvious in classical female patients and late-onset male and female patients (see Figure S2, S3 in supplementary material).

**Table 3:** Comparison of renal involvement in patients with classical Fabry disease at different sexes and ages

	Males (n = 152)				Females (n = 53)			
	Preschool group (n = 0)	Juvenile group (n = 38)	Young group (n = 111)	Middle-aged / elderly group (n = 3)	Preschool group (n = 2)	Juvenile group (n = 9)	Young Group (n = 27)	Middle-aged / elderly group (n = 15)
**Proteinuria**	/	13/38 (34.21)	90/111 (81.08) ^**b**^	2/3 (66.67)	0/2	2/9 (22.22)	8/27 (29.63)	4/15 (26.67)
**Renal insufficient**	/	5/38 (13.16)	64/111 (57.66) ^**b**^	1/3 (33.33)	0/2	1/9 (11.11)	1/27 (3.70)	4/15 (26.67)
**ESRD**	/	0/38	34/111(30.63) ^**b**^	0/3	0/2	0/9	0/27	1/15 (6.67)

Abbreviations: ESRD, end-stage renal disease; Note: ^**b**^ Compared with the juvenile group, there is a statistical difference (*P* < 0.05)

In this study, 37 patients had started hemodialysis or undergone kidney transplantation (37/232, 15.94%), including 36 males and 1 female, 35 patients with classical phenotype and 2 patients with late-onset phenotype. 33 patients received hemodialysis, with more males than females (16.49% vs. 1.05%, *P* < 0.05), and the mean age of classical patients at the initiation of hemodialysis was 37.10 ± 5.03 years. Four patients underwent kidney transplantation. Of the 33 patients who received dialysis, 10 underwent kidney transplantation after hemodialysis.

In this study, 75 patients (24.12%) received renal biopsy, and the renal histomorphological changes were similar. Under light microscope, vacuolar degeneration of glomerular podocytes was observed, some glomerular loops adhered to the cyst wall, and even segmental sclerosis occurred. Under electron microscope, osmiophilic and concentric myeloid bodies of different sizes were observed in various renal cells.

## Discussion

This study is the largest clinical study of Fabry disease in China to date. First, the results of this investigation showed that patients with Fabry disease had a long delay of more than ten years from the onset of the initial manifestations to the final diagnosis. Regarding the age at manifestation onset, age at the first medical specialty visited and age at the diagnosis of Fabry disease, males were earlier than females, and males with classical phenotype were earlier than males with late-onset and females with classical phenotype. In both male and female patients, the initial manifestations of classical patients were mainly acroparesthesia, and the first medical specialty visited were mainly pediatrics and neurology. The initial manifestations of late-onset patients were mainly renal and cardiovascular involvement, and the first medical specialty visited were mainly nephrology and cardiology. Second, in classical patients, both male and female, the initial manifestations of the preschool and juvenile groups were mainly acroparesthesia, and the frequency of renal and cardiovascular involvement in young group was higher than that in preschool and juvenile groups. Third, more than 50% of males with classical Fabry disease develop proteinuria of varying degrees at age 25 and renal insufficiency at age 40. 15.94% of the patients progressed to dialysis or kidney transplantation, mainly classical males. These results suggest that clinicians, especially pediatrics, neurology, nephrology and cardiology, should consider the possibility of Fabry disease in preschoolers and juveniles who present with acroparesthesia and hypohidrosis/anhidrosis as well as in young, middle-aged and elderly patients who have renal or cardiovascular (especially adult females) involvement.

In this study, the mean ages of Fabry disease patients at the onset of the initial manifestations and the first visit were 14.69 and 19.63 years, respectively, and the mean age at final diagnosis was 29.55 years. The mean interval between the onset of the first clinical manifestations and the final diagnosis was approximately ten years. Meanwhile, the number of patients with classical Fabry disease was relatively high (76.21%), and only 23.79% had late-onset. Among them, the age at manifestation onset, age at first medical specialty visited and age at diagnosis in patients with the classical phenotype were significantly earlier than those in patients with the late-onset phenotype, and the enzyme activity of patients with the classical phenotype was significantly lower than that of patients with the late-onset phenotype, which was consistent with previous research results [14]. The enzyme activity of classical patients will be significantly decreased or completely absent, mostly in male patients, often in childhood onset, early symptoms can appear acroparesthesia, anhidrosis or hypohidrosis, cornea vortex turbidity, and other symptoms, adult can progress to renal, cardiovascular and nervous system involvement. However, in patients with late-onset phenotype, the enzyme activity is partially decreased or normal, most of which occur in adults, often in women, and often only manifested as one organ involvement, such as the heart or kidney. Due to the lack of specificity of clinical manifestations and the existence of different clinical phenotypes in patients with Fabry disease, it is easy to misdiagnose. Studies [9, 18] have reported that in Europe and the United States, the mean interval from the onset of the initial manifestations to the final diagnosis of Fabry disease is 13.7–16.3 years. Studies [19] in eastern China have also reported that the interval from the onset of manifestations to the diagnosis of Fabry disease is approximately 14.8 years, and the longest interval can be decades, which is basically consistent with the results of this study. The most common initial manifestation of classical Fabry disease patients in this study was acroparesthesia, followed by hypohidrosis or anhidrosis and renal involvement. Among them, acroparesthesia is an early, common and important clinical manifestation of Fabry disease. It has been reported that more than 75% of patients will have neuropathic limb pain in the early stage, and the mean age of onset is approximately 10 years [20–22]. Pain in the extremities often manifests as pain in the distal ends of the extremities; this pain can be aggravated by weather changes, fever, mental stress or exercise; and this pain is often accompanied by fever and heat intolerance [20, 21, 23]. Due to the deposition of enzyme metabolizing substrate in the skin, sweat gland secretion dysfunction and atrophy of sweat glands and sebaceous glands, ischemic injury caused by stenosis of blood vessels supplying sweat glands, and sweat gland denervation caused by autonomic nervous dysfunction, patients with Fabry disease usually suffer from perspiration obstruction in summer, with systemic or localized hypoperspiration [24, 25]. Since general auxiliary examinations (such as routine laboratory examination and imagological examination) show no specific changes and differences between individuals are large, patients will be transferred to different hospitals and medical specialties according to their chief complaints. Furthermore, due to clinicians’ lack of understanding of this disease, especially those in primary hospitals, most of them diagnose other more common clinical diseases, such as growth pain, rheumatic pain, primary erythromelalgia, Raynaud’s syndrome, or fibromyalgia [9, 10]. Previous studies in Europe and the United States [18, 26] have reported that approximately 64% of patients with Fabry disease will be misdiagnosed as having other diseases. Therefore, early and accurate diagnosis and treatment to delay the progression of the disease and prevent progressive multiorgan failure over the natural course of the disease have become extremely important key factors in the diagnosis and treatment of Fabry disease. This also prompts the inclusion of Fabry disease in the differential diagnosis when multisystem involvement is encountered in the clinic.

More than half of the patients in this study were tested for *GLA* gene and enzyme activity, and genetic testing is the gold standard for disease diagnosis. In this study, missense mutations accounted for the largest proportion of the variation in the *GLA* gene of Fabry disease, which was basically consistent with previous studies [27]. Meanwhile, the geographic spread throughout China could be greatly affected by issues such as medical infrastructure and medical knowledge/awareness of this genetic disorder. The results of this study may not necessarily reflect the real distribution differences of the epidemic areas.

Previous clinical studies of Fabry disease have mostly focused on the clinical manifestations, diagnosis and treatment. There are few reports on how the initial manifestations of classical and late-onset patients evolve with sexes and ages. In this study, in classical patients, both male and female, the initial manifestations of the preschool and juvenile groups were mainly acroparesthesia, and the frequency of renal and cardiovascular involvement in young group was higher than that in preschool and juvenile groups. The frequency and severity of acroparesthesia tended to decrease with age, while the frequency of renal and cardio/cerebrovascular involvement increased. Due to the small sample size of patients with late-onset, especially after being dispersed to different sexes and age groups, the sample size of each group was even smaller, and there was no significant statistical difference in results. It has been reported [28, 29] that children and minors suffer mainly from pain and show less cardiac and renal involvement. However, as the disease progresses in adults, multiorgan involvement is exacerbated, especially in the kidneys and the cardio/cerebrovascular systems. Most classical male patients will develop ESRD in the third to fifth decade of life. This is important because damage to important organs, such as the kidneys and heart, occurs after manifestations such as extremity pain, which also provides a potential treatment opportunity for intervening and changing the disease course of patients diagnosed in childhood. Early diagnosis and treatment before the important target organs of the heart, brain and kidneys are not involved is of great clinical importance for alleviating manifestations and reducing the risk of life-threatening complications. Meanwhile, this study also found that the incidence of adult female patients with cardiovascular involvement as the initial manifestation and cardiology as the first visit medical specialty was significantly higher than that of males. However, there are few clinical studies on the cardiovascular system of female patients with Fabry disease, and further discussion is needed. The findings of this study showed that the pediatric, neurology, and nephrology were the first visited and the final diagnosis medical specialties of patients. Preschool and juvenile patients mostly visit the pediatric and neurology medical specialties for limb pain, and young, middle-aged and elderly patients visit the nephrology medical specialty for renal involvement. These findings suggest that pediatricians and neurologists should consider the possibility of Fabry disease in preschoolers and juveniles who present with acroparesthesia. Furthermore, these findings remind nephrologists to consider the possibility of Fabry disease renal involvement when young, middle-aged and elderly patients have proteinuria and renal insufficiency. In adult female patients with unexplained cardiovascular involvement, clinicians should also be vigilant for the possibility of Fabry disease, and corresponding detection should be conducted to clarify the diagnosis and reduce missed diagnosis and misdiagnosis.

Progressive kidney disease is one of the main manifestations of Fabry disease with multiorgan involvement. Clinically, more than half of classical male patients and more than 20% of female patients will eventually develop different degrees of renal involvement and even progress to ESRD. Renal failure is also a common and important late complication and the main cause of death due to Fabry disease [30, 31]. Among them, proteinuria is one of the early manifestations of renal involvement in patients with Fabry disease, and it is also an important risk factor for the progression of renal disease [14, 32, 33]. Studies [34] have reported that approximately 50% of untreated men with classic Fabry disease will develop proteinuria of varying degrees by the age of 35 years and chronic renal insufficiency by the age of 42 years. The incidence of proteinuria in men increases with age, reaching approximately 90% by the age of 50. Furthermore, as the disease progresses, patients will develop progressive renal insufficiency and eventually ESRD. In addition, approximately 30-35% of females with Fabry disease develop significant proteinuria, but the onset is usually later than that of males [9, 35]. This study focused on analyzing the evolution of renal involvement in classical and late-onset Fabry patients of different sexes and ages. In classical patients, there was no obvious renal involvement in either male or female patients at preschool age. Renal involvement was most common in young patients. More than 50% of male classical patients had varying degrees of proteinuria and chronic renal insufficiency at the ages of 25 and 40, respectively.

Since the deposition of enzyme metabolic substrates in renal tissue is responsible for the renal manifestations of the disease, renal biopsy may be helpful in the diagnosis of Fabry disease, especially in patients with genetic variants of unknown significance, those with mild manifestations or those who are asymptomatic. In this study, 75 patients (24.12%) with Fabry disease underwent renal biopsy. Light microscopic analysis of renal biopsy samples from patients with Fabry disease with renal involvement mainly shows extensive and diffuse vacuolar degeneration of glomerular podocytes [34]. Electron microscopic analysis shows a large number of “onion skin-like” or “zebra-like” osmophilic inclusions with a diameter of approximately 1–3 μm in the cytoplasm of various cells in the kidneys, especially podocytes and renal tubular epithelial cells. These inclusions are myeloid bodies, and they are an important basis for the diagnosis of the disease [36], but secondary factors such as the use of amiodarone or hydroxychloroquine sulfate and Niemann Pick disease should be excluded in clinical practice. [37].

ESRD is a common complication of Fabry disease, and some patients, especially classical male patients, eventually start dialysis or undergo kidney transplantation due to progression to ESRD. In a long-term natural history study of 105 male patients with Fabry disease from the National Institutes of Health (NIH), 23% of patients with Fabry disease eventually progressed to ESRD [34]. Approximately 50.51% of Fabry disease patients in this study had family members with ESRD, and approximately 15.94% of Fabry disease patients received renal replacement therapy. The mean age of classical male patients with Fabry disease at the start of dialysis was approximately 37 years, which is basically consistent with previous research reports. Several studies reported that the average age of ESRD male patients with Fabry disease was 35–43 years [34, 38, 39]. In addition, the prevalence of Fabry disease in the dialysis population ranges from 0.02–1.2% [34, 40–44].

This study did not explore the relationship between the initial manifestations and clinical prognoses of patients with Fabry disease, nor did it explore the biological mechanism underlying the development of the initial manifestations in patients. In addition, most patients included in this study had classic Fabry disease and were conscious of their manifestations, and their manifestations were mainly subjective discomfort manifestations. There are two groups of patients less represented in this study, including patients with late-onset and patients detected by eye and ear examination. However, studies from Europe and the United States have reported that late-onset disease is more common [45]. Therefore, it is necessary to pay more attention to the screening and diagnosis of late-onset Fabry disease in the Chinese population to reduce the rate of miss diagnosis of the disease. In addition, it is necessary to further explore the enzyme activity and genetic variation of Fabry disease and the correlation between enzyme activity or gene variation and clinical phenotype to better guide the diagnosis and evaluation of the disease.

## Electronic supplementary material

Below is the link to the electronic supplementary material.

Supplementary Material 1

## Data Availability

The datasets used in this study are available from the corresponding author on reasonable request.

## Abbreviations

α-Gal A: Alpha-galactosidase A
Gb3: Globotriaosylceramide
Lyso-Gb3: Lyso-globotriaosylceramide
ERT: Enzyme replacement therapy
ESRD: End-stage renal disease
