# Supplementary material for: The evolution of the initial manifestations and renal involvement of chinese patients with classical and late-onset Fabry disease at different sexes and ages
Source: BMC Nephrol. 2023 Apr 5;24:90. doi: 10.1186/s12882-023-03138-w (PMC10074707; doi:10.1186/s12882-023-03138-w)
Supplement: Supplementary file 1 — Supplementary Material 1 [file 12882_2023_3138_MOESM1_ESM.docx]

**Table S1. Comparison of the initial manifestations of late- onset patients with Fabry disease at different sexes and ages [N=35, n/N (%)]**

|  | **Males（n=15）** | | | | | | | **Females（n=20）** | | | |
| --- | --- | --- | --- | --- | --- | --- | --- | --- | --- | --- | --- |
|  | **Preschool group**  **(n=1)** | **Juvenile**  **group**  **(n=2)** | **Young**  **group**  **(n=12)** | **Middle-aged / elderly group (n=0)** | | | | **Preschool group**  **（n=2）** | **Juvenile**  **group**  **(n=6)** | **Young**  **group**  **(n=9)** | **Middle-aged / elderly group (n=3)** |
| **Acroparesthesia (limb pain)** | 0/1 | 0/2 | 0/12 | | / | | | 0/2 | 0/6 | 0/9 | 0/3 |
| **Anhidrosis/ hypohidrosis** | 0/1 | 1/2 (50.00) | 0/12 | | | / | | 1/2 (50.00) | 2/6 (33.33) | 0/9 ^a, b^ | 0/3 ^b^ |
| **Renal invo** | 0/1 | 1/2 (50.00) | 11/12 (91.67) ^a^ | | | / | | 1/2 (50.00) | 1/6 (16.67) | 4/9 (44.44) | 2/3 (66.67) |
| **Cardiovascular invo** | 0/1 | 0/2 | 1/12 (8.33) | | | | / | 0/2 | 0/6 | 4/9 (44.44) | 1/3 (33.33) |
| **Angiokeratomas** | 0/1 | 0/2 | 0/12 | | | | / | 0/2 | 0/6 | 0/9 | 0/3 |
| **Neuropsychiatric invo** | 0/1 | 0/2 | 0/12 | | | | / | 0/2 | 2/6 (33.33) | 1/4 (25.00) | 0/3 ^b^ |
| **Digestive system invo** | 1/1 (100) | 0/2 | 0/12 | | | | / | 0/2 | 0/6 | 0/9 | 0/3 |
| **Others manifestations** | 0/1 | 0/2 | 0/12 | | | | / | 0/2 | 0/6 | 0/9 | 0/3 |

Abbreviations: invo, involvement; Note: **^a^** Compared with the preschool group, there is a statistical difference (*P*<0.05); **^b^** Compared with the juvenile group, there is a statistical difference (*P*<0.05)


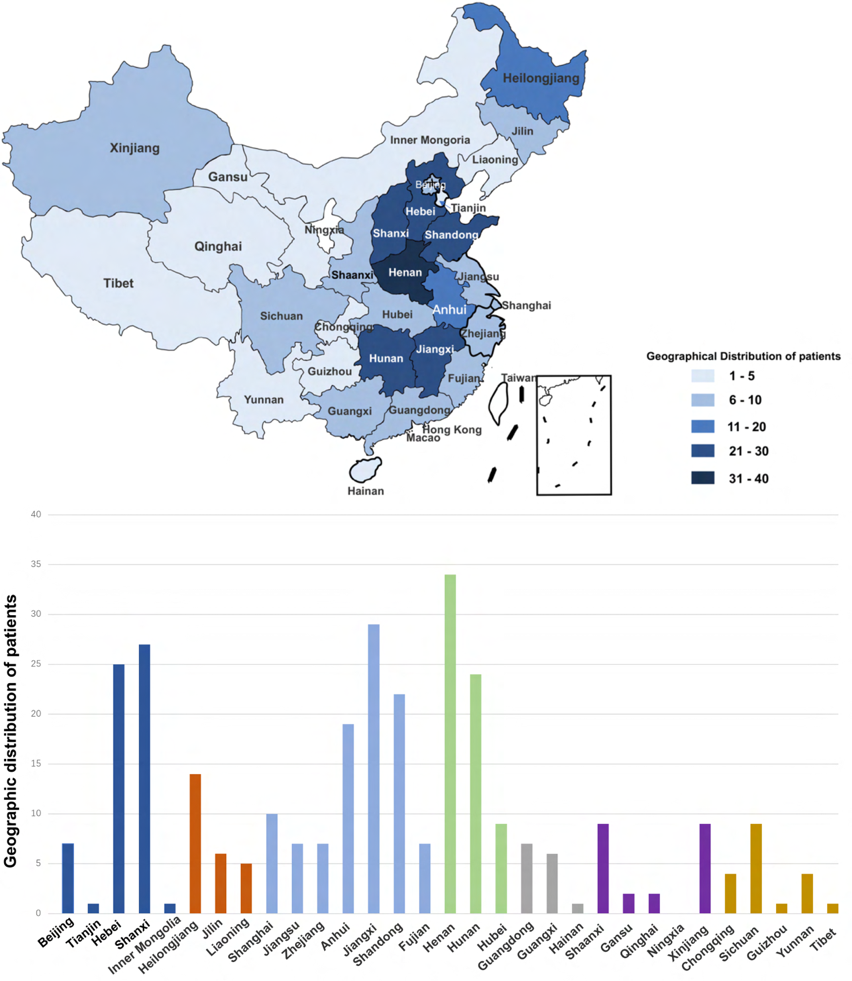


**Figure S1. Geographical distribution of patients with Fabry disease in China.**

Fabry disease patients in China have spread across 30 provinces and autonomous regions, and were mainly concentrated in East, Central and North China, among which Henan accounted for the most.


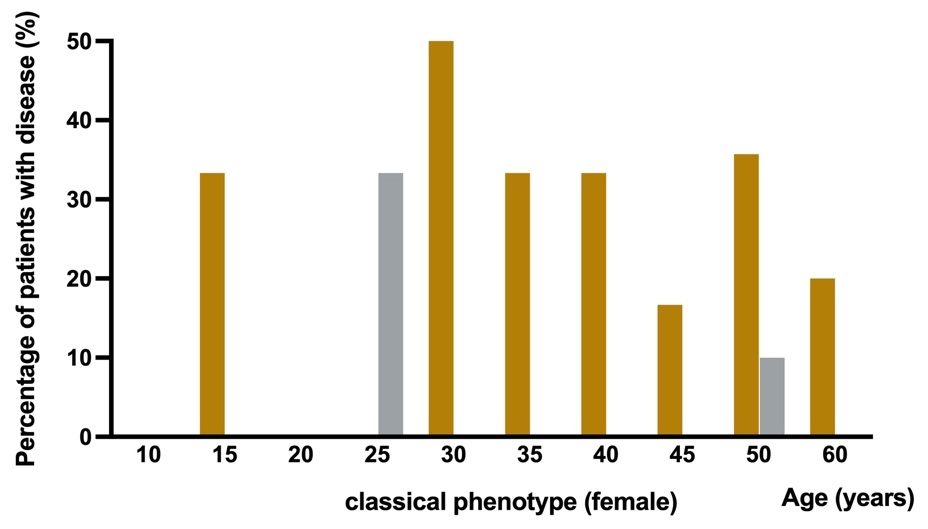


**Figure S2.** **Evolution of renal involvement in classical female Fabry disease patients with aging.** The evolution of proteinuria and renal insufficiency in classical female patients has no obvious regularity, and the renal involvement occurs mostly in young and middle-aged groups.


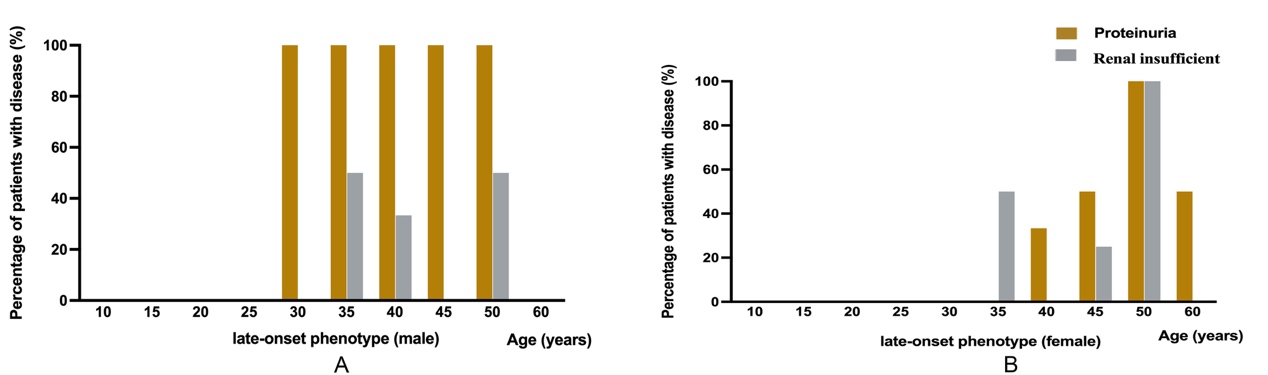
 **Figure S3. Evolution of renal involvement in late-onset male and female Fabry disease patients with aging.** Due to the small sample size of male and female patients with late-onset (13 males and 14 females), the results were biased, and there was no obvious regularity in the evolution of renal involvement with aging.
